# Analysis of Osteocalcin Hormone Proteoforms with Vitamin D Deficiency in Patients with Diabetes Mellitus Using Multiple-Reaction Monitoring–Mass Spectrometric Immunoassay Approach

**DOI:** 10.3390/mps9040117

**Published:** 2026-08-12

**Authors:** Refat M. Nimer, Hicham Benabdelkamel, Afshan Masood, Maha Al Mogren, Salini Scaria Joy, Anas M. Abdel Rahman, Assim A. Alfadda

**Affiliations:** 1 Department of Medical Laboratory Sciences, Faculty of Applied Medical Sciences, Jordan University of Science and Technology, Irbid 22110, Jordan; rmnimer@just.edu.jo; 2 Proteomics Resource Unit, Obesity Research Center, College of Medicine, King Saud University, Riyadh 11461, Saudi Arabia; afsmasood@ksu.edu.sa (A.M.); aalfadda@ksu.edu.sa (A.A.A.); 3 Metabolomics Section, Precision Medicine Laboratory Department, Genomic Medicine Center of Excellence (GMCoE), King Faisal Specialist Hospital and Research Centre (KFSHRC), Riyadh 11211, Saudi Arabia; mmogren@kfshrc.edu.sa; 4 Strategic Center for Diabetes Research, College of Medicine, King Saud University, Riyadh 11461, Saudi Arabia; sjoy@ksu.edu.sa; 5 Department of Biochemistry and Molecular Medicine, College of Medicine, Alfaisal University, Riyadh 11533, Saudi Arabia; 6 Department of Medicine, College of Medicine, King Saud University, Riyadh 11461, Saudi Arabia

**Keywords:** type 2 diabetes mellitus, vitamin D deficiency, mass spectrometric immunoassay (MSIA), multiple reaction monitoring, osteocalcin

## Abstract

Osteocalcin (OC), a bone protein, is evaluated by its proteoforms, which are implicated in various illnesses. Rapid, clinically used immunoassays are well-established. Epitope dependence, fragment cross-reactivity, uneven identification across carboxylation states, and lack of standardization restrict their efficacy. This study aims to characterize various OC proteoforms in patients with type 2 diabetes mellitus (T2DM) and vitamin D insufficiency and to develop a mass spectrometric immunoassay (MSIA) for their detection. The first MRM-MSIA test to specifically detect OC proteoforms in plasma with vitamin D deficiency-induced alterations in T2DM patients was developed and validated. A precise and selective MRM-MSIA was developed to assess OC fragments in plasma from T2DM patients with normal (DN) or deficient (DD) vitamin D levels. The approach examined OC-1 (aa64–71), OC-2 (aa64–70), and OC-3 (aa72–93) proteoforms. It met international linearity, precision, and accuracy criteria with variability <15% and intra/inter-day accuracy of 88.8% to 110.1%. The DD group had significantly higher OC-1 levels than the DN group. The receiver operating characteristic (ROC) curve for OC-1 produced an AUC of 0.8263 (95% confidence interval [CI], *p* = 0.0004). The developed MRM-MSIA measures plasma OC proteoforms with good specificity and sensitivity, making it a promising clinical diagnostic tool.

## 1. Introduction

Osteocalcin (OC), also known as bone gamma-carboxyglutamic acid (Gla) protein (BGP), is a 5.7 kDa peptide comprising 49 amino acids. It is synthesized by osteoblasts and is the most abundant noncollagenous protein in the bone matrix [1]. OC is initially produced as a 98-amino-acid pre-pro-protein, which undergoes proteolytic processing to yield the mature form. This mature OC, called carboxylated OC (cOC), binds strongly to bone and the extracellular matrix. It undergoes γ-carboxylation at three glutamic acid residues (positions 17, 21, and 24) by the enzyme γ-glutamyl carboxylase. Alongside vit D (vit D), OC was originally postulated to function in extracellular matrix mineralization and has been employed as a circulating biomarker of osteoblastic bone formation [2,3].

In recent years, studies have highlighted the involvement of both vit D and OC in insulin resistance and the development of metabolic diseases such as type 2 diabetes mellitus (T2DM) [4,5,6]. Uncarboxylated OC (ucOC) has been shown to stimulate insulin secretion from pancreatic islets and plays a key role in glucose homeostasis [7].

Deficiency of vit D is common worldwide, particularly among individuals suffering from T2DM. Recent studies have shown that more than 50% of patients with T2DM are suffering from vit D deficiency [8,9]. Furthermore, vit D adequacy is important for better glycemic control, particularly in vulnerable populations or individuals who have vitamin D deficiency. These findings highlight the strong link between low vit D status and glucose metabolism, and the significance of the investigation of vit D-based biomarkers and the interrelation between vit D and OC in diabetes [10].

OC levels have been associated with serum 25-hydroxyvitamin D [25(OH)D] concentrations, which are frequently reduced in individuals who are overweight or at elevated risk for developing T2DM [11]. Vit D status is typically classified as deficient when levels are <50 nmol/L, insufficient between 50 and 70 nmol/L, and sufficient when >75 nmol/L [12].

The γ-carboxylated residues of OC are essential for calcium binding. However, under the acidic conditions of osteoclast resorption compartments, OC undergoes decarboxylation, reducing its bone-binding affinity and promoting the release of ucOC into the bloodstream [13]. In addition to the intact protein, multiple OC fragments are present in the bloodstream [14]. Vit D directly increases OC gene transcription via a vit D responsive element, while vitamin K regulates its γ-carboxylation [15].

Numerous commercial immunoassays have been developed to quantify circulating OC levels. These include radioimmunoassay (RIA), enzyme-linked immunosorbent assay (ELISA), electrochemiluminescence immunoassays (ECLIA), chemiluminescent immunoassays (CLIA). These tests are commonly used in clinical settings because they are easy to use, fast, and efficient [16,17,18]. However, significant variability arises from preanalytical factors, the lack of international standardization, and the coexistence of both intact OC (amino acids 1–49) and its fragments, particularly the stable N-terminal/mid-region (N-MID) fragment (amino acids 1–43). The full-length OC molecule is relatively unstable due to cleavage between amino acids 43 and 44. Although the N-MID fragment is more stable, conventional immunoassays lack the specificity to distinguish between cOC and ucOC [19]. However, these methods are insufficient for detailed proteoform analysis, particularly under metabolic conditions such as T2DM, underscoring the need for a more robust, proteoform-specific approach. Undercarboxylated OC plays an important role in the regulation of insulin secretion in pancreatic beta-cells, adiponectin expression in adipocytes, insulin sensitivity, and energy balance [20].

Given the potential role of OC proteoforms in glucose metabolism and their modulation by vit D status, characterizing these forms may provide insights into diabetes pathophysiology and support the development of improved diagnostic tools.

Therefore, developing and validating robust analytical methods is required to accurately quantify known OC variants and to examine additional proteoforms.

In clinical and routine practice, OC is usually analyzed using immunoassays that measure total OC without distinguishing the proteoforms [21]. These assays have limitations and do not measure specific OC proteoforms for metabolic disorders. Mass spectrometry-based assays for the analysis of 25-hydroxyvitamin D, steroid hormones, and parathyroid hormone have entered clinical use due to their higher specificity and lower interference [22,23,24]. The liquid chromatography–mass spectrometry method (LC-MS) may be used as an additional approach and as a possible replacement for the future of immunoassays with enhanced specificity, increased speed, expanded analyte coverage, greater throughput, multiplexing capacity, lower cost per sample, and reduced sample volume requirements [25]. At the moment, no clinically validated mass spectrometry assay is available for measuring OC proteoforms, creating a clear gap for testing. In order to address this gap, we validated a multiple reaction monitoring–mass spectrometric immunoassay (MRM-MSIA) assay to identify circulating OC proteoforms in subjects with T2DM based on their vitamin D status.

Mass spectrometric immunoassays (MSIA) have emerged as powerful tools for biomarker analysis, providing exceptional specificity and sensitivity [26]. MSIA combines the target-binding potential of immunoassays with the precision of mass spectrometry (MS). The first MSIA, developed by Nelson et al. in 1995 using matrix-assisted laser desorption/ionization (MALDI)-MS, enabled the identification of myotoxin αI via antibodies conjugated to agarose beads [27]. MSIA allows for microscale capture, identification, and quantification of target proteins. Clinically important proteoforms such as apolipoprotein A-I, apolipoprotein C-III, insulin-like growth factor 1, and amyloid A have been successfully measured using validated MSIA methods [28,29,30,31]. There is a relationship between vit D and pancreatic β-cell function. In this case, the progression of prediabetes has been related to vitamin D and its inhibitory effect on pathological progression [32]. This is because vitamin D has demonstrated potential to increase the likelihood of a normal metabolic state, an essential factor in the progression of diabetes [32]. Moreover, reports indicate that vit D reduces the risk of diabetes development by up to 15%, especially when it exceeds the normal limit for bone health [33]. Thus, the validated MSIA approach allows specific detection and characterization of distinct OC proteoforms, thereby potentially providing additional pathophysiological insight. This study aimed to characterize the various OC proteoforms in relation to vit D insufficiency in patients with T2DM and to describe an MSIA specifically developed for the identification and quantification of OC proteoforms.

## 2. Materials and Methods

### 2.1. Materials and Chemicals

The anti-osteocalcin monoclonal antibody (OCG4, Cat. No. MA1-20788), modified Dulbecco’s phosphate-buffered saline (PBS, Cat. No. 28344), and PBS with Tween-20 (PBST, Cat. No. 28346) were purchased from Thermo Fisher Scientific, Waltham, MA, USA. Protein A/G 96 tips (MSIA D. A. R. T.; Cat. No. 991PRT15) and Finnipipette Novus multichannel electronic pipettes were also obtained from Thermo Scientific. All reagents and chemicals, including acetonitrile (ACN), methanol (MeOH), formic acid (FA), dithiothreitol (DTT), iodoacetamide (IAA), and ammonium bicarbonate (ABC), were purchased from Sigma-Aldrich (St. Louis, MO, USA). Both stable isotope-labeled (^13^C_6_, ^15^N_2_-labeled lysine and ^13^C_6_, ^15^N_4_-labeled arginine) and their unlabeled counterparts were synthesized by Biomatik Corporation (Kitchener, ON, Canada).

### 2.2. Ethical Considerations

All procedures adhered to the ethical principles outlined in the Declaration of Helsinki and followed the guidelines established by the International Conference on Harmonization for Good Clinical Practice (ICH-GCP) [34,35]. The study was approved by the Institutional Review Board (IRB) of the College of Medicine, King Saud University (Approval No: E-16-1752). Written informed consent was obtained from all participants before their enrollment in the study.

### 2.3. Subjects and Sample Collection

A total of 40 patients with T2DM receiving primary care at the outpatient clinics of King Saud University Medical City were enrolled. No participants had comorbidities related to cardiovascular disease, chronic kidney disease, liver disease, thyroid disorders, active infections, malignancy, or autoimmune diseases. Blood samples were collected into EDTA tubes (BD Biosciences, San Jose, CA, USA) after a 10 h overnight fast. Plasma was separated by centrifugation at 3000× *g* for 15 min and stored at −80 °C for later analysis. Routine laboratory investigations and estimations of 25 (OH) Vit D levels were carried out at the central biochemistry lab at King Khalid University Hospital, Riyadh, Saudi Arabia. The 25(OH)D levels were measured using electrochemiluminescence (ECLIA) immunoassay (Modular Analytics E170, Roche Diagnostics GmbH, Mannheim, Germany). Patients were assessed for their vit D status and categorized accordingly. Patients with serum vit D levels >75 nmol/L were classified as having T2DM with normal vit D levels (DN, *n* = 20), whereas those with levels <50 nmol/L were categorized as having T2DM with deficient vit D levels (DD, *n* = 20). Characteristics of study participants are presented in Table S1.

### 2.4. Selection of Signature Peptides

The full-length protein sequence of OC from Homo sapiens (accession number P02818) was retrieved from the National Center for Biotechnology Information (NCBI) protein database. To identify potential signature peptides suitable for targeted proteomics, the sequence was subjected to in silico trypsin digestion using the PeptideMass tool (https://web.expasy.org/peptide_mass/, accessed 12 August 2024) available at the ExPASy Bioinformatics Resource Portal. Peptide specificity was confirmed using the Basic Local Alignment Search Tool (BLAST) (https://ftp.ncbi.nlm.nih.gov/blast/, accessed 12 August 2024) by comparing the sequences against other human proteins in the NCBI database. Several criteria were applied to select optimal signature peptides. First, peptides with 5–25 amino acids were considered for efficient ionization and detection by LC-MS/MS. Second, water solubility was evaluated using PepCalc (https://pepcalc.com/, accessed 12 August, 2024Innovagen). Third, peptide stability was assessed using the ProtParam tool (http://web.expasy.org/protparam/, accessed 12 August 2024). Fourth, selected peptides were verified as OC-specific using the SIM alignment tool (https://web.expasy.org/sim/, accessed 12 August 2024) to prevent cross-reactivity. Finally, potential post-translational modifications (PTMs), including methylation, glycosylation, and phosphorylation, were screened using UniProt and Phospho.ELM databases. Regions containing known γ-carboxylated glutamate residues were evaluated during peptide selection. Peptides containing these labile or variable post-translational modification sites were excluded to ensure analytical robustness and reproducibility in the MRM assay.

Characteristic OC peptides were identified via in silico tryptic digestion and subsequently confirmed using Skyline software v23.1. Table S2 summarizes the features of the selected OC-specific signature peptides used for quantification. Light and heavy peptide pairs served as analytes and internal standards. Each peptide pair co-eluted at identical retention times, ensuring reliable quantification. Calibration curves constructed from standard reference materials demonstrated consistent ionization efficiencies between the unknown samples and calibration standards.

### 2.5. MRM Transition Development

The candidate OC proteoforms were identified from LC-MS/MS data by analyzing their intact mass and fragmentation patterns. These were then aligned with recommended practices for proteoform identification and validation [36,37]. The selection was further guided by the known biological processing of OC, as documented in protein databases such as Uniprot, and by the presence or absence of PTMs on OC, specifically the γ-carboxylation and truncation variants, which are well-known [38,39]. From the different proteoforms present, only those supported by reproducible spectral data, precise mass measurements, and confident sequence assignment, adhering to current proteoform reporting standards, were retained [40]. Features lacking sufficient confidence or clear proteoform specificity were excluded from further analysis.

#### 2.5.1. Preparation of Synthetic Peptides

Unlabeled and stable-isotope-labeled peptides corresponding to each OC proteoform were first prepared as stock solutions at a concentration of 1.0 mg/mL in water. For LC-MS/MS analysis, a working solution of each peptide was prepared by diluting the stock to a final concentration of 10.0 µg/mL in the mobile phase, which consisted of solvent A (0.1% FA in distilled water) and solvent B (0.1% FA in 50% (*v*/*v*) ACN: MeOH).

#### 2.5.2. Liquid Chromatography–Mass Spectrometry (LC-MS/MS) Analysis

Each peptide was analyzed at a final concentration of 10.0 µg/mL using a Waters Acquity UPLC coupled to an XEVO TQ-MS operated in positive electrospray ionization (ESI) mode. Chromatographic separation employed a UHPLC BEH C18 column (1.7 µm, 2.1 × 100 mm) at a flow rate of 0.3 mL/min [41]. The gradient program started with 90% solvent A and 10% solvent B for 1 min, followed by a linear gradient over 10 min to reach 10% solvent A and 90% solvent B. The gradient then returned to its initial condition over 3 min, resulting in a total run time of 15 min. For LC-MS/MS, adjustments were made to the overall mass spectrometry (MS) settings. MS parameters included a desolvation temperature of 450 °C, desolvation gas flow of 900 L/h, cone gas flow of 50 L/h, cone voltage of 40 V, and capillary voltage of 2 kV.

The MRM transitions for each OC peptide and their respective internal standards were optimized and chosen based on signal strength and specificity. The specific transitions used were as follows: OC-1 (327.32 > 183.133) and its labeled internal standard (417.37 > 282.16); OC-2 (412.42 > 272.194) and its labeled internal standard (417.37 > 282.16); OC-3 (837.9 > 253.1) and its labeled internal standard (840.2 > 253.02) (Table S2). These transitions were selected for their signal intensity and fragmentation patterns to enable precise quantification of the targeted proteoforms.

### 2.6. Sample Preparation and Enrichment Using MSIA D.A.R.Ts

**Figure 1:**
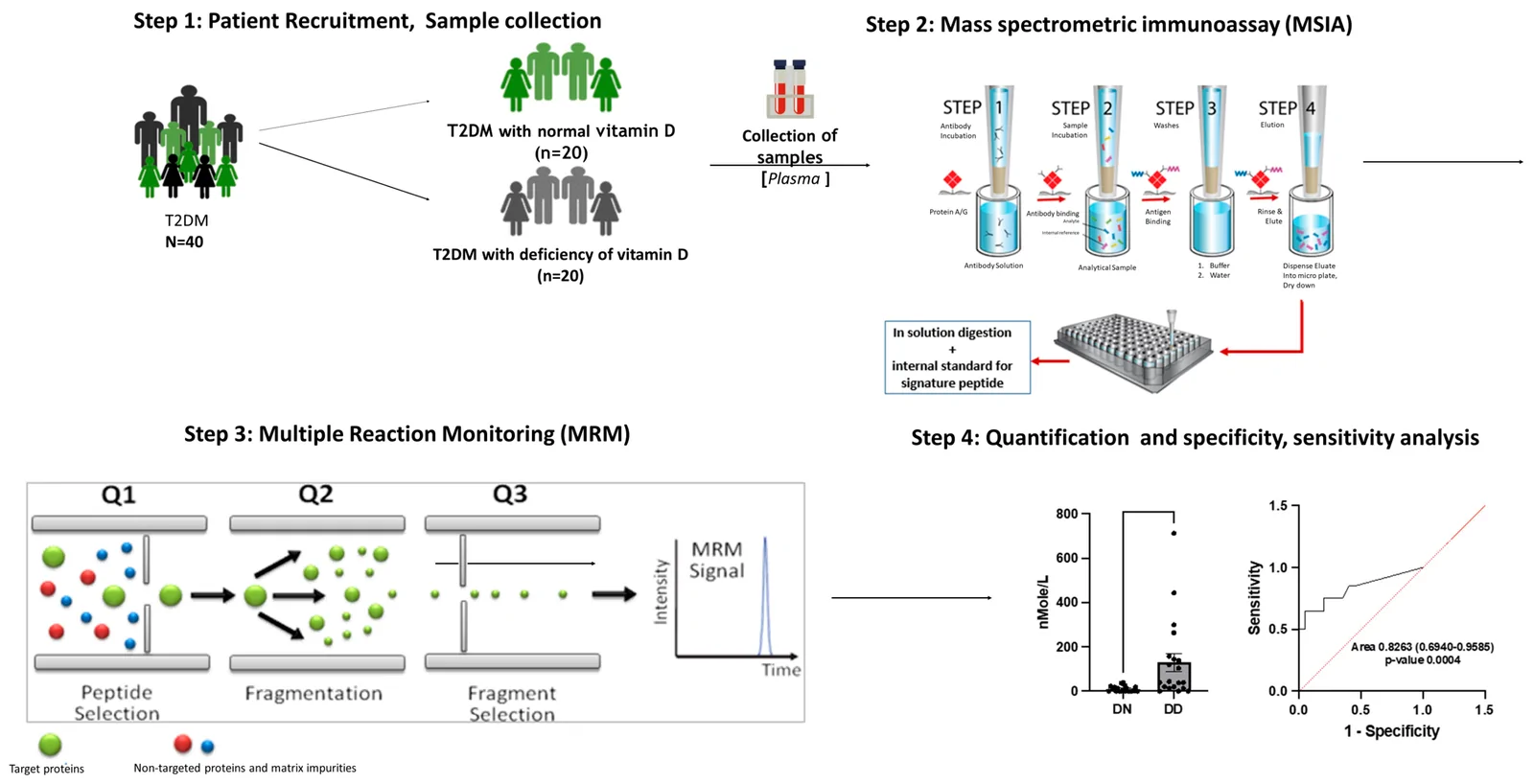
Experimental protocol for osteocalcin mass spectrometric immunoassay.

Plasma samples were processed using MSIA D.A.R.Ts (Thermo Scientific, Waltham, MA, USA) according to the manufacturer’s protocol to isolate the target OC proteoforms. Novus i multichannel pipettes equipped with protein A/G MSIA™ tips were used to facilitate sample enrichment through repeated aspiration and dispensing cycles (Table S3). For each sample, plasma was diluted 10-fold in PBST. Then, 15 μL of the diluted plasma was mixed with 135 μL of 100× PBS, and the mixture was applied to the conditioned MSIA tips. Before applying the sample, the tips were preconditioned with PBS and then loaded with 100 μL of anti-OC polyclonal antibody (0.05 mg/mL), which binds OC at epitopes 1–100. The antibody was immobilized via its interaction with protein A/G on the MSIA monolithic surface. Antigen–antibody binding enabled the selective extraction of native OC proteoforms. Four wash steps (alternating between PBS and water) were performed to remove any unbound components. Bound OC proteoforms were eluted using a buffer containing 40% ACN and 0.4% TFA in distilled water. The eluted samples were then dried using a high-speed vacuum centrifuge and stored until LC-MS/MS analysis. A schematic of the overall MSIA workflow for IC is shown in Figure 1. Immunoaffinity capture using antibody-coated porous tips is shown in Figure S1.

#### 2.6.1. In-Solution Tryptic Digestion

Tryptic digestion was performed by dissolving proteins in 40 μL of 50 mM ammonium bicarbonate and incubating the solution at 56 °C for 1 h with 10 mM dithiothreitol (DTT; Sigma, St. Louis, MO, USA) to reduce disulfide bonds. Proteins were then alkylated with 20 mM iodoacetamide (IAA; Sigma, St. Louis, MO, USA) at room temperature in the dark for 1 h. Subsequently, enzymatic digestion was carried out using trypsin (Promega, Madison, WI, USA) at a 1:50 (*w*/*w*) enzyme-to-substrate ratio, followed by overnight incubation at 37 °C. The reaction was terminated by adding 7 μL of 10% FA.

#### 2.6.2. Solid-Phase Extraction (SPE)

Tryptic peptides were purified and concentrated using solid-phase extraction (SPE), as described previously [42]. Oasis HLB 1 cc 30 mg cartridges (Waters Oasis PRiME, Waters Corporation, Milford, MA, USA) were conditioned with 100% MeOH, followed by equilibration with distilled water. Peptide samples, premixed with stable isotope-labeled internal standards and diluted in 0.1% FA in distilled water (loading buffer), were then loaded onto the SPE cartridges. Unbound substances were removed by two washes with distilled water. Finally, peptides were eluted using 1 mL of 50% ACN containing 0.1% FA.

### 2.7. Validation of the LC-MS/MS Method

The validation of the analytical method was conducted in accordance with the guidelines set by the United States Food and Drug Administration (US FDA) and the International Conference on Harmonization (ICH) [43,44,45,46]. Sample analysis utilized the LC-MS/MS method outlined in Section 2.5.2. All validation experiments adhered to predefined acceptance criteria and the number of replicates (*n*), which are detailed in the validation tables. The assay performance was summarized using standard statistical measures such as mean, SD, %CV, and % bias, as appropriate. For LC-MS validation, precision was considered acceptable at ≤15% CV and accuracy within ±15% bias. At the lower limit of quantitation (LLOQ), acceptable limits were ≤20% CV and ±20% bias. These criteria were consistently applied to ensure a transparent and reproducible evaluation of the assay’s performance.

#### 2.7.1. Linearity

A calibration curve was created using unlabeled peptides at concentrations from 0.1 to 1000 nM. Quality control (QC) samples were prepared at 1.5, 35, and 75 nM. Proteins from pooled plasma samples were precipitated by adding four volumes of ice-cold acetone, vortexing for 1 min, and incubating at −20 °C for 1 h. After centrifuging at 14,000× *g* for 15 min at 4 °C, the supernatant was discarded, and the pellet was air-dried. The pellets were resuspended in PBS and digested with trypsin to prepare the matrix. Calibration standards and QC samples were then made by spiking synthetic peptides into this digested matrix. Before injection, each sample was diluted with 0.1% formic acid in deionized water and spiked with 20 µL of a 20 µg/mL internal standard prepared in 0.1% formic acid in a 50:50 (*v*/*v*) mixture of acetonitrile and methanol, then processed as described in Section 2.5.2. Calibration curves were built over three consecutive days, plotting the ratio of analyte to internal standard signal (y-axis) against the nominal concentration (x-axis). Linearity was evaluated through regression analysis based on the correlation coefficient (R^2^), as well as precision and accuracy. Calibration curves were evaluated over three independent analytical runs (*n* = 3). Accuracy and precision acceptance criteria were ±15% bias and ≤15% CV, respectively.

#### 2.7.2. Specificity and Sensitivity

Calibration curves collected over three consecutive days were used to determine the lower limit of detection (LLOD) and LLOQ. The LLOD and LLOQ were defined as concentrations with signal-to-noise (S/N) ratios of at least 3 and 10, respectively. The LLOD and LLOQ were calculated as follows: LLOD = 3.3σ/S while LLOQ = 10σ/S, where σ represents the standard deviation of the response, and S represents the slope of the calibration curve. The LLOQ was needed to demonstrate accuracy within 80–120% and precision (coefficient of variation) of 20% or less.

Specificity was evaluated by confirming that the detected signals were uniquely linked to the targeted OC proteoforms using precise mass measurements, consistent retention times, and confirmatory MS/MS fragmentation. Additionally, potential interference from endogenous background, co-eluting species, and related peptide/proteoform forms was assessed. Control and blank samples were checked to ensure that no significant interfering signals were present at the relevant retention times and *m*/*z* ranges.

#### 2.7.3. Inter- and Intraday Validation

Precision and accuracy were evaluated at three quality control (QC) levels: low (QCL), medium (QCM), and high (QCH) using six replicate injections on the same day at concentrations of 35, 150, and 300 nM, respectively. Intraday (within-day) precision and accuracy were evaluated based on these replicates, while interday (between-day) reproducibility was determined by analyzing the same QC levels over three consecutive days under identical experimental conditions. Precision was expressed as % relative standard deviation (%RSD). Intraday accuracy was calculated as

[(mean estimated concentration on a given day)/(nominal concentration)] × 100

Interday accuracy was calculated as

[(mean concentration over 3 days)/(nominal concentration)] × 100

Acceptable accuracy criteria were defined as 80–120% for the LOQ calibrators and 85–115% for all other calibration levels.

#### 2.7.4. Peptide Stability

QC samples (at high, medium, and low concentrations) were stored at room temperature, 4 °C, and −20 °C to evaluate peptide stability. After storage, these samples were analyzed and compared to freshly prepared QC samples. Peptide stability was calculated as [(mean area ratio of stored sample)/(mean area ratio of fresh sample)] × 100%.

#### 2.7.5. Carryover

Carryover was evaluated by injecting three blank samples following the injection of the highest standard concentration, and this step was repeated six times to ensure consistency. Carryover was considered acceptable if a signal detected in the blank samples did not exceed 20% of the LLOQ [47].

### 2.8. Data and Statistical Analysis

GraphPad Prism 6.01 (La Jolla, CA, USA) was used to perform statistical comparisons of clinical variables between groups. A *p*-value < 0.05 was considered statistically significant. LC-MS/MS data acquisition and processing were performed using MassLynx software version 4.1 (Waters Corporation, Milford, MA, USA). All quantitative results are reported as mean ± standard deviation (SD). OC1-3 proteoform levels were compared between patients with T2DM associated with vit D deficiency and those with normal vit D levels.

## 3. Results

### 3.1. Participant Characteristics

All participants had T2DM and were classified as either DN (normal vit D) or DD (vit D deficient). The mean ages of the DN and DD groups were 50.3 ± 13.2 years and 45.3 ± 10.6 years, respectively. No significant differences were observed between the groups in age or biochemical parameters (*p* > 0.05), whereas vit D levels were significantly different (*p* < 0.01).

### 3.2. Characteristics of Signature Peptides

Three signature peptides, ^64^PDPLEPRR^71^, ^64^PDPLEPR^70^, and ^72^EVCELNPDCDELADHIGFQEAY^93^, were selected based on the criteria in Section 2.4. These peptides were synthesized in both unlabeled and stable isotope-labeled forms. These were used to construct calibration curves and served as peptides generated in both regular and stable-isotope-labeled forms as internal standards (IS). All peptides had a purity > 95% (confirmed by HPLC) and underwent sequence confirmation. High-resolution mass spectrometry (HRMS) was employed to verify isotopic labeling sites. The manufacturer verified the quality of the standard peptide through MS spectra and HPLC chromatograms.

### 3.3. Method Validation Results

#### 3.3.1. Establishment of Linear Curve

**Figure 2:**
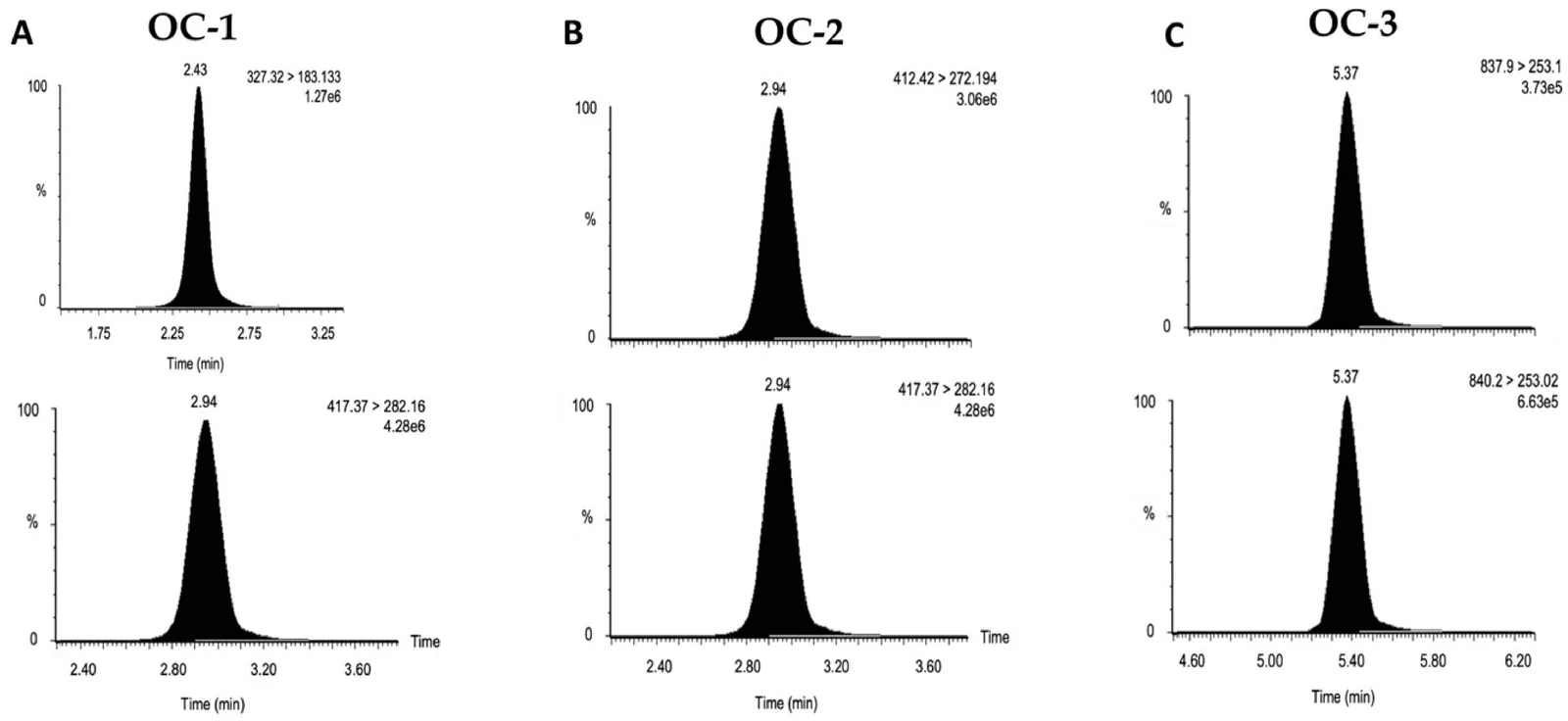
Extracted ion chromatograms (EIC) showing transitions of osteocalcin (OC) variants and their corresponding internal standard transitions, analyzed at a concentration of 10 µg/mL: (**A**) Quantitative transitions for OC-1 (327.32 > 183.133) and its labeled internal standard (417.37 > 282.16); (**B**) Quantitative transitions for OC-2 (412.42 > 272.194) and its labeled internal standard (417.37 > 282.16); (**C**) Quantitative transitions for OC-3 (837.9 > 253.1) and its labeled internal standard (840.2 > 253.02).

**Figure 3:**
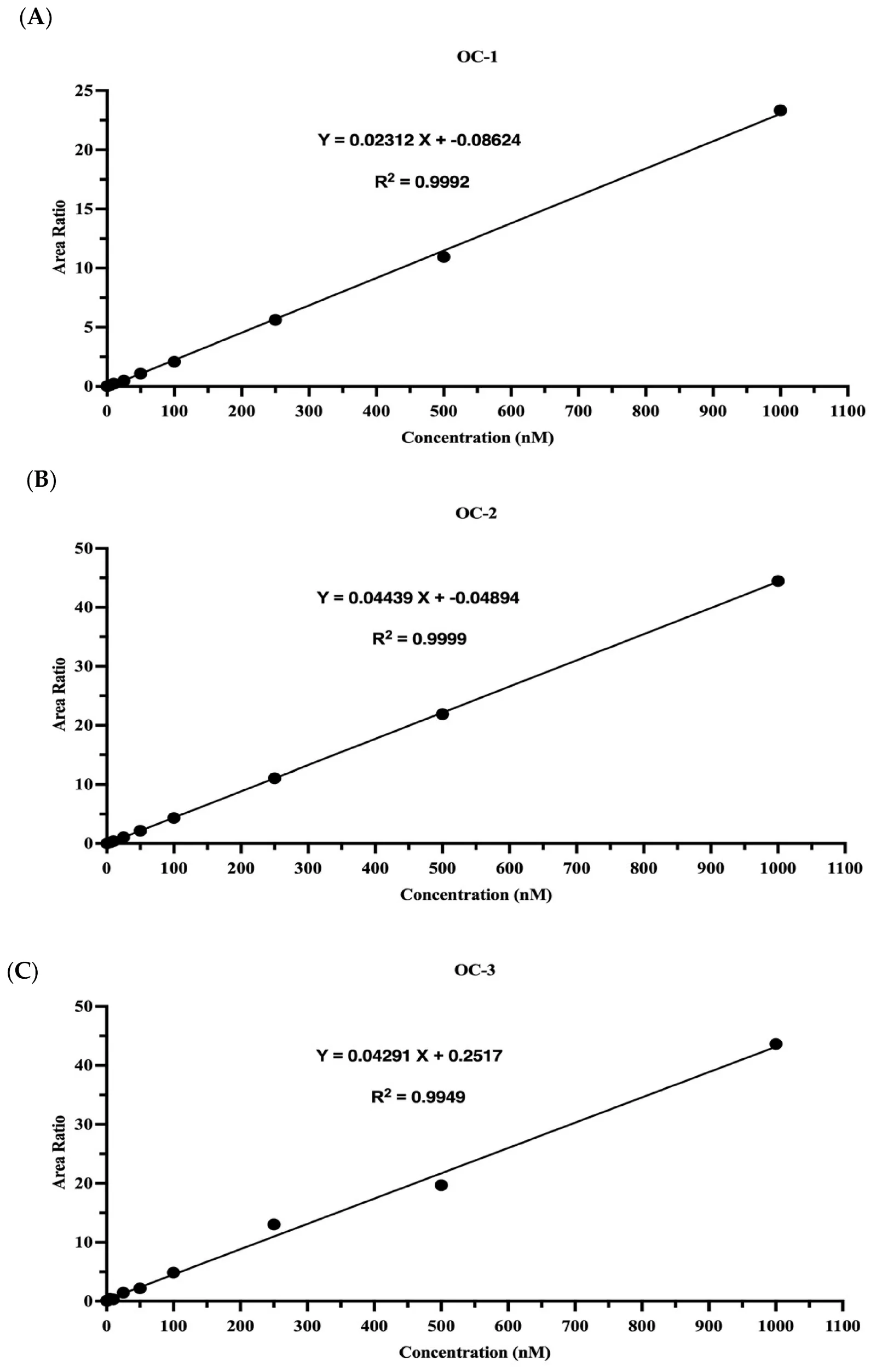
Representative calibration curves for (**A**) OC-1, (**B**) OC-2, and (**C**) OC-3, where the x-axis represents the nominal concentration of the proteoform in nM, and the y-axis represents the area ratio between the unlabeled-to-labeled signature peptide of each proteoform measured.

The quantification range and linearity for each selected OC signature peptide (aa64–71, aa64–70, and aa72–93) were established using calibration curves. The calibration curves demonstrated excellent linearity over the range of 0.5 to 1000 nM, with coefficients of determination (R^2^) of 0.9992, 0.9999, and 0.9949, respectively (Figure 2 and Figure 3). Table 1 shows the results of the sensitivity evaluation. The calibration curves for the three OC peptides had coefficients of determination (R^2^) > 0.99, confirming accurate quantification across a broad concentration range (0.5 to 1000 nM). In addition, no significant interfering signals were observed in blank or control samples at the retention times and monitored MRM transitions corresponding to the targeted OC proteoforms, confirming the specificity of the assay.

The LLOQ was 0.5 nM for the characteristic peptides, confirmed over three days. Calibration curves for the three signature OC peptides demonstrated high linearity with R^2^ values of 0.9993, 0.9998, and 0.9950. No significant interference was observed in specificity testing, and blank samples showed signals below 20% of the LLOQ.

The attained LLOQ of 0.5 nM for all three OC peptides demonstrates adequate sensitivity for detecting physiological concentrations of OC fragments in plasma. Circulating OC fragment levels typically range from approximately 0.9 to 3.9 ng/mL, corresponding to ~0.16–0.76 nM, depending on assay methodology and population studied. These values often fall within the sub-nanomolar to low-nanomolar range, especially for certain OC proteoforms [48]. Consequently, the sensitivity of our technique allows accurate measurement of low-abundance osteocalcin fragments, underscoring its appropriateness for clinical diagnostic applications and proteoform-specific biomarker investigations. Analytically, the MSIA approach offers sensitivity comparable to that of the enzyme-linked immunosorbent assay (ELISA) [49]; however, it provides significantly enhanced specificity for proteoform-resolved quantification of OC fragments.

#### 3.3.2. Inter- and Intraday Precision

Intra- and interday variability was less than 15%, and accuracy ranged from 88.8% to 110.1% (Table 2 and Table 3), meeting ICH and FDA acceptance criteria. These results indicate minimal analytical variability and support the method’s reproducibility over multiple runs and days.

#### 3.3.3. Peptide Stability Study

Stability testing showed peptide integrity ranging from 81.3% to 107.5% (Table S4). This suggests that the method is not only analytically reliable but also feasible for routine use in clinical laboratories, where samples may be subjected to varying storage and handling conditions.

### 3.4. Evaluation of OC Variants in Clinical Samples

**Figure 4:**
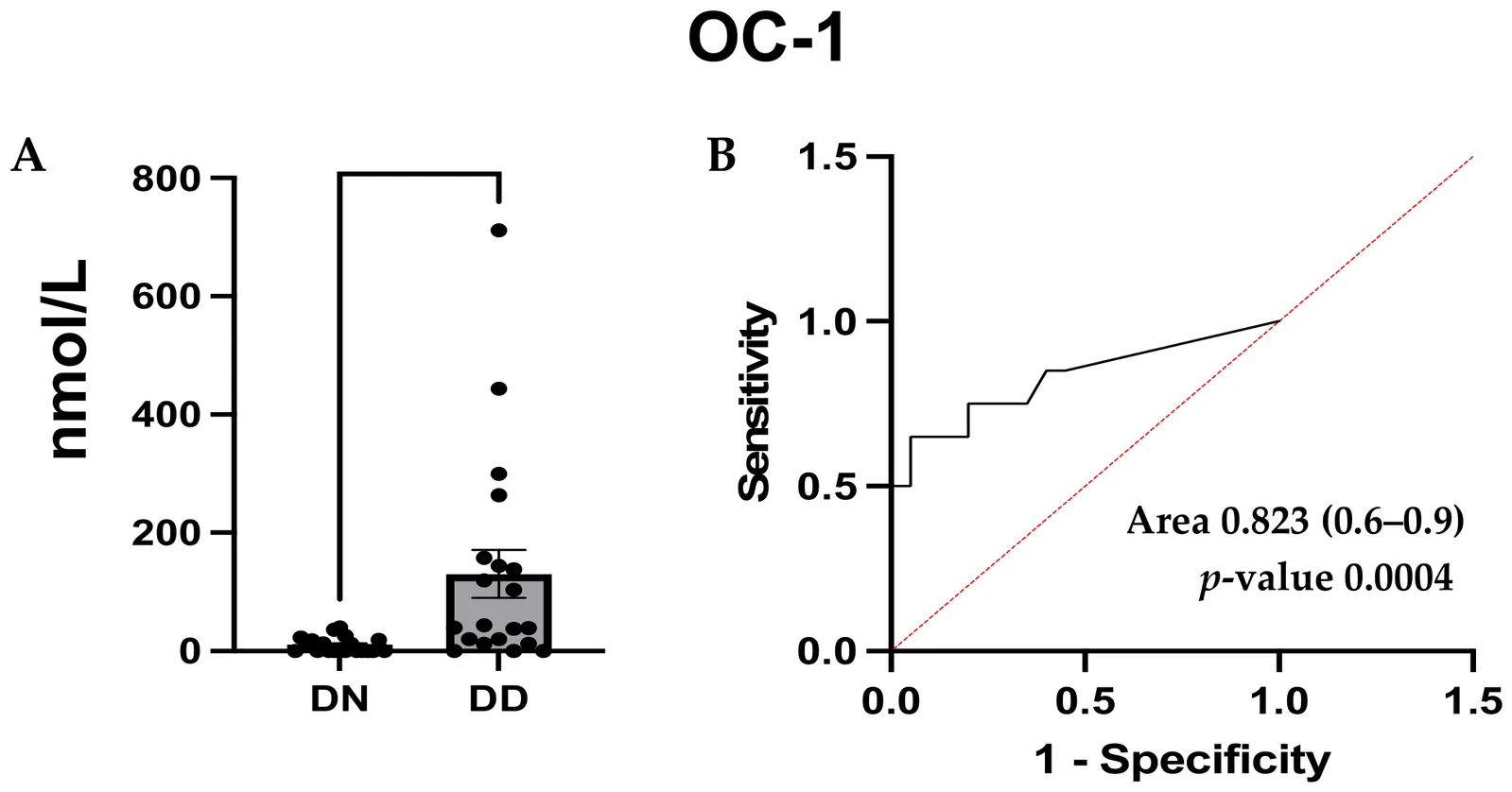
(**A**) Comparison of OC-1 (aa64–71) levels between subjects with T2DM and vit D insufficiency vs. those with normal vit D levels. A significant elevation in OC-1 was observed in the vit D insufficient group (*p* ≤ 0.01); (**B**) ROC curve evaluating the diagnostic performance of OC-1 in patients with T2DM and vit D insufficiency in comparison to those with T2DM and normal vit D levels. The area under the curve (AUC) for OC-1 was 0.8263 (95% CI, *p* = 0.0004). The diagonal reference red line represents no discriminatory ability (AUC = 0.50), corresponding to classification performance expected by chance.

Significantly elevated levels of OC-1 (aa64–71) were observed in patients with T2DM and vit D deficiency compared to those with normal vit D levels (*p* ≤ 0.01) (Figure 4A). The between-group comparison for the remaining proteoforms OC-2 and OC-3 showed no significant difference between the two groups. The ROC curve analysis for OC-1 yielded an AUC of 0.8263 (95% confidence interval [CI], *p* = 0.0004) (Figure 4B).

## 4. Discussion

Different OC variants have distinct clinical roles. Among the known variants that are commonly measured using immunoassays are the carboxylated and uncarboxylated OC (ucOC). Carboxylated OC is a key marker of bone health, supporting bone strength and mineralization, and ucOC is known to influence metabolic processes such as glucose and energy metabolism. Higher ucOC levels correlate with increased insulin sensitivity and a lower incidence of T2DM [50]. OC levels also monitor bone remodeling in diseases like osteoporosis, Paget’s disease, and chronic renal disease, guiding clinical management [48,51,52]. Changes in OC carboxylation and calcium coordination markedly affect its structure and biological functions [53]. Vit D deficiency is known to lower OC gene expression in osteoblasts, resulting in reduced blood OC levels [54]. It also impairs calcium absorption, which may affect OC activation [55]. Guney et al. found a positive correlation between OC and vit D levels in patients with metabolic syndrome [56]. Vit D supplementation, on the other hand, is known to increase OC levels, while decreasing ucOC levels [57]. These data suggest that vit D levels modulate the balance of OC proteoforms.

Traditional antibody-based methods for biomarker measurement are limited by their inability to differentiate proteoforms and polymorphic variants in a single assay and often face issues with stability, sensitivity, and cost [58]. Recent advances include biosensors, chemiluminescent immunoassays, and LC-MS/MS techniques that improve specificity and sensitivity [21,58,59] and identify these different proteoforms.

A previous study reported successful quantification of parathyroid hormone fragments using MRM-MSIA, demonstrating the method’s specificity and sensitivity [60]. This study is the first to apply MSIA for reliable detection and quantification of OC fragments in normal vit D and patients with T2DM, validating this approach.

OC plays a central role at the intersection of bone metabolism and systemic glucose homeostasis. It acts as a hormone, influencing insulin secretion, insulin sensitivity, and overall glucose regulation. Vitamin K-dependent carboxylation converts osteocalcin into the carboxylated form that binds hydroxyapatite and supports mineralization, and the carboxylation status has been associated with type 2 diabetes in recent population studies [61]. On the other hand, ucOC exhibits endocrine activity and is the active form in glucose metabolism. It enhances insulin secretion, β-cell proliferation, and insulin sensitivity stimulating insulin secretion potentially improving insulin sensitivity in muscle and adipose tissue [62]. Similarly, vit D is essential for OC synthesis and carboxylation in osteoblasts, and its deficiency may disrupt these processes, leading to altered levels and functionality of circulating osteocalcin. In diabetes, impaired bone turnover and oxidative stress further affect osteocalcin expression and processing [63]. The mechanistic basis for a vitamin D influence on osteocalcin is reinforced by interventional evidence. In a randomized, double-blind trial in patients with type 2 diabetes mellitus, supplementation with vitamin D3 significantly reduced circulating undercarboxylated osteocalcin and lowered the undercarboxylated-to-carboxylated ratio, whereas vitamin K2 raised the carboxylated form [64]. This indicates that vitamin D status actively shapes the balance of osteocalcin species rather than merely correlating with total concentrations, and it provides a plausible framework for interpreting the altered osteocalcin profile observed in our vitamin D–deficient cohort. The combination of vit D deficiency and diabetes creates a unique biochemical environment that shifts OC proteoform balance, potentially influencing both bone remodeling and metabolic regulation. Our findings of altered OC-1 levels in this context may reflect such underlying pathophysiological changes. This study demonstrates, for the first time, the application of MSIA-LC-MS/MS to detect and quantify OC proteoforms in T2DM patients with vit D deficiency, offering high specificity and sensitivity over traditional immunoassays that can only distinguish between carboxylated and undercarboxylated OC. These findings suggest the potential utility of OC proteoform profiling in assessing vit D status and metabolic dysfunction in T2DM.

The observed elevation of the proteoform OC-1 (aa64–71) in vit D-deficient T2DM patients may reflect a shift in osteocalcin processing, likely influenced by dysregulated bone remodeling. One possible explanation is enhanced proteolytic cleavage at the C-terminal region, potentially mediated by vit D-regulated enzymes such as matrix metalloproteinases or cathepsin K. Alternatively, OC-1 may exhibit greater biochemical stability or resistance to further degradation compared to other overlapping fragments, contributing to its increased abundance. The findings from our study may suggest that vit D deficiency may alter the balance of OC proteoforms, with potential implications for bone–metabolism cross-talk in metabolic disease.

Key strengths of the study include the use of stable isotope-labeled peptides, rigorous validation using standard reference materials, and peptide-level quantification, which addresses limitations of antibody cross-reactivity. However, limitations include a relatively small sample size, lack of longitudinal data, the absence of formal evaluation of matrix effects and recovery, and the requirement for specialized equipment and technical expertise, which may constrain broader clinical adoption. Limitation of MRM include that accuracy can be impacted by factors such as the quality of peptide selection, digestion efficiency, sample processing consistency, and potential interference from matrix effects or co-eluting compounds. MSIA is also limited by challenges such as experimental complexity, low signal intensity in MS, and the restricted availability of high-quality antibodies [65]. Future studies involving larger, diverse cohorts and therapeutic monitoring, such as vit D supplementation, are warranted to further validate clinical applicability and explore the prognostic value of specific OC proteoforms in metabolic and bone disorders.

## 5. Conclusions

The multiple reaction monitoring–mass spectrometric immunoassay (MRM-MSIA) assay has proven to be useful in the quantitation of OC proteoforms as it demonstrated good analytical performance characterized by low LLOD and LLOQ, good precision, reproducibility, stability, and recovery. The results highlight the substantial potential of OC proteoforms as potential biomarkers for monitoring in patients with T2DM and vit D deficiency. Additionally, further investigation is warranted to establish a definitive relationship between vit D supplementation and the distribution of OC proteoforms.

## Figures and Tables

**Table 1:** Linearity and sensitivity summary over three days.

Signature Peptide ID	LLOQ (*n* = 6)			Slope (*n* = 3)	Intercept (*n* = 3)	R^2^ (*n* = 3)
	Concentration (nM)	Inter-Day Accuracy (%)	Inter-Day Precision (%)			
OC-1	0.50	98.7	7.1	0.02311	−0.08630	0.9993
OC-2	0.50	105.3	2.9	0.04440	−0.04890	0.9998
OC-3	0.50	101.3	4.1	0.04289	0.2520	0.9950

OC, osteocalcin; LLOQ, Lower Limit of Quantification; R^2^, coefficient of determination.

**Table 2:** Inter-day validation summary.

Signature Peptide ID	QCL 35.0 nM (*n* = 6)				QCM 150.0 nM (*n* = 6)				QCH 300.0 nM (*n* = 6)			
	Mean (nM)	SD	Accuracy %	Precision %	Mean (nM)	SD	Accuracy %	Precision %	Mean (nM)	SD	Accuracy %	Precision %
OC-1	34.9	1.57	99.71	4.5	155.2	2.6	103.47	1.70	330.3	22.0	110.1	6.7
OC-2	33.4	1.06	95.4	3.17	154.07	8.16	102.7	5.3	310.3	4.7	103.4	1.5
OC-3	35.2	2.65	100.57	7.5	158.9	9.4	105.9	5.9	311.3	14.2	103.7	4.6

QCL, Quality Control Low; QCM, Quality Control Medium; QCH, Quality Control High; SD, standard deviation; OC, osteocalcin.

**Table 3:** Intra-day validation summary.

Signature Peptide ID	QCL 35.0 nM (*n* = 18)				QCM 150.0 nM (*n* = 18)				QCH 300.0 nM (*n* = 18)			
	Mean (nM)	SD	Accuracy %	Precision %	Mean (nM)	SD	Accuracy %	Precision %	Mean (nM)	SD	Accuracy %	Precision %
OC-1	35.2	3.9	100.7	11.0	153.2	7.7	102.1	5.0	308.5	17.5	102.8	5.7
OC-2	31.0	2.7	88.8	8.9	159.2	5.0	106.1	3.1	298.5	15.2	99.5	5.1
OC-3	31.6	2.0	90.2	6.3	147.5	11.4	98.3	7.7	303.2	16.4	101.1	5.4

QCL, Quality Control Low; QCM, Quality Control Medium; QCH, Quality Control High, SD, standard deviation, OC, osteocalcin.

## Data Availability

The datasets generated, used, and/or analyzed during the current study are available from the corresponding author upon reasonable request.

## Supplementary Materials

The following supporting information can be downloaded at: https://www.mdpi.com/article/10.3390/mps9040117/s1, Table S1: Characteristics of study participants. Table S2: List of osteocalcin signature peptides and their internal standard details, including the instrumental conditions. Table S3: Outlines the pipetting technique for OC purification using a Thermo Finnpipette Novus i Multichannel Electronic Pipette equipped with Protein A/G MSIA-tips. Table S4: Summary of osteocalcin peptides stability under various storage environments for a specified duration. Figure S1: Immunoaffinity capture using antibody-coated porous tips.

## Abbreviations

The following abbreviations are used in this manuscript:

25(OH)D	25-hydroxyvitamin D
ABC	ammonium bicarbonate
ACN	acetonitrile
BLAST	Basic Local Alignment Search Tool
cOC	carboxylated OC
DTT	dithiothreitol
EIC	Extracted ion chromatograms
FA	formic acid
HRMS	High-resolution mass spectrometry
IAA	iodoacetamide
ICH-GCP	International Conference on Harmonization for Good Clinical Practice
IRB	Institutional Review Board
LC-MS/MS	Liquid Chromatography–Mass Spectrometry
LOD	lower limit of detection
LLOQ	lower limit of quantitation
(MALDI)-MS	matrix-assisted laser desorption/ionization mass spectrometry
MeOH	methanol
MRM-MSIA	multiple reaction monitoring–mass spectrometric immunoassay
MS	mass spectrometry
MSIA	mass spectrometric immunoassay
NCBI	National Center for Biotechnology Information
N-MID	N-terminal/mid-region
OC	Osteocalcin
PC	post-translational modifications
QC	Quality control
QCH	Quality Control High
QCL	Quality Control Low
QCM	Quality Control Medium
SPE	solid-phase extraction
T2DM	type 2 diabetes mellitus
ucOC	uncarboxylated OC
Vit D	vitamin D
