# Supplementary material for: Analysis of Osteocalcin Hormone Proteoforms with Vitamin D Deficiency in Patients with Diabetes Mellitus Using Multiple-Reaction Monitoring–Mass Spectrometric Immunoassay Approach
Source: Methods Protoc. 2026 Aug 12;9(4):117. doi: 10.3390/mps9040117 (PMC13515257; doi:10.3390/mps9040117)
Supplement: Supplementary file 1 [file mps-09-00117-s001.zip › mps-4400348-supplementary.pdf]

Table S1: Characteristics of study participants.

Parameter	Vitamin D Deficiency (DD) (n = 20)	Normal Vitamin D (DN) (n = 20)	*p*-Value
Male/Female (%)	60/40	70/30	NA
Age (years)	45.3 ± 10.6	50.3 ± 13.2	0.12
Glucose (mmol/L)	6.3 ± 0.8	7.2 ± 0.5	0.09
Urea (mmol/L)	4.3 ± 0.9	4.6 ± 0.8	0.26
Creatinine (umol/L)	71.1 ± 12.7	75.3 ± 12.0	0.07
Sodium (mmol/L)	132.6 ± 5.1	136.3 ± 5.9	0.13
Potassium (mmol/L)	4.2 ± 0.2	4.3 ± 0.4	0.40
Aspartate transaminase (IU/L)	44.8 ± 9.1	41.8 ± 4.5	0.38
Alanine transaminase (IU/L)	19.5 ± 5	18.8 ± 2.0	0.4
Alkaline phosphatase (IU/L)	97.6 ± 13.7	93.2 ± 28.9	0.3
Total cholesterol (mmol/L)	4.6 ± 1.0	4.9 ± 0.8	0.10
HDL cholesterol (mmol/L)	1.2 ± 0.2	1.4 ± 0.2	0.2
LDL cholesterol (mmol/L)	2.8 ± 0.9	3.1 ± 0.7	0.09
Triglycerides (mmol/L)	1.8 ± 0.6	2.5 ± 0.2	0.07
vitamin D (nmol/L)	27.7± 9.3	97.5 ± 22.9	<0.01 *

Values are presented as mean ± SD. The Student's t test is used to compare the means between two groups.
* $p < 0.05$ considered statistically significant.

Table S2: List of osteocalcin signature peptides and their internal standard details, including the instrumental conditions.

No.	Peptide sequence	Molecular weight (g/mol)	Q1 (m/z) [z]	Q3 (m/z)	Product ion type [z]	Type	Cone voltage (V)	CE (V)	Retention time (min)	Quantification Property
OC-1	^{64}PDPLEPRR^{71}	979.2	327.32 [3]	183.13	B5 [3]	Standard	25	20	2.43	Quant
				175.09	Y1 [1]		25	20	2.43	Qual
OC-2	^{64}PDPLEPR^{70}	822.9	412.42 [2]	213.05	B2 [1]	Standard	26	15	2.96	Qual
				272.18	Y2 [1]		26	25	2.96	Quant
	^{64}PDPLEPR^{70}	832.9	417.37 [2]	213.04	B2 [1]	Internal	26	25	2.96	Quant
				282.16	Y2 [1]	Standard	26	25	2.96	Qual
OC-3	^{72}EVCELNPDCDELADHIGFQEAY^{93}	2510.7	837.9 [3]	182.12	Y1 [1]	Standard	34	25	5.39	Qual
				253.16	Y2 [1]		34	20	5.39	Quant
	^{72}EVCELNPDCDELADHIGFQEAY^{93}	2517.7	840.2 [3]	182.12	Y1 [1]	Internal	34	25	5.39	Qual
				253.02	Y2 [1]	Standard	34	20	5.39	Quant

OC, osteocalcin; Q1; Precursor Ion m/z; Q3; Product Ion m/z, CE; Collision Energy

Table S3: outlines the pipetting technique for OC purification using a Thermo Finnpipette Novus i Multichannel Electronic Pipette equipped with Protein A/G MSIA -tips.

	**1 Wash**	**2 Immobilization of Antibody**	**3 Wash**	**4 Antigen capture**	**5 Wash**	**6 Wash**	**7 Wash**	**8 Wash**	**9 Elution**
**Liquid**	PBS	100ul (0.05 mg/mL) of the Mouse anti Osteocalcin Hormone (OC) antibody	10 mM PBS	150uL of 100x diluted plasma in PBST	10 mM PBS	10 mM PBS	LC/MS grade Water	LC/MS grade Water	40% acetonitrile and 0.4% TFA LC/MS grade Water
**Asp/Disp cycles**	10x	250x	10x	100x	10x	10x	10x	10x	10x
**Cycle volume (uL)**	150	80	150	100	150	150	150	150	7
**Total well volume (uL)**	200	100	200	100	200	200	200	200	10

OC, osteocalcin; PBS, Phosphate-Buffered Saline; PBST, Phosphate-Buffered Saline with Tween; TFA, Trifluoroacetic Acid

Table S4. Summary of osteocalcin peptides stability under various storage environments for a specified duration

Signature Peptide ID	QC (nM)	Stability (%)							
		Fresh (-80 °C)		RT (24 h)		4°C (1 Week)		–20 °C (1 Month)	
		Mean (%)	SD	Mean (%)	SD	Mean (%)	SD	Mean (%)	SD
OC-1	35.0	100	2.1	93.3	7.5	81.1	10.2	81.3	2.4
	150.0	100	5.7	92.7	4.7	91.3	11.5	90.4	1.3
	300.0	100	7.8	90.5	2.3	95.7	5.1	94.1	5.2
OC-2	35.0	100	2.3	89.0	3.2	102.4	9.3	96.0	5.3
	150.0	100	3.4	97.9	6.8	105.6	2.9	89.1	1.4
	300.0	100	7.8	101.6	8.6	107.5	11.8	92.2	3.4
OC-3	35.0	100	6.9	89.3	3.6	81.7	10.0	94.7	4.1
	150.0	100	9.6	91.2	1.2	93.9	11.0	83.0	1.7
	300.0	100	8.0	88.5	2.6	94.9	10.7	88.1	12.2

QC; Quality Control, RT; Room Temperature, OC, osteocalcin

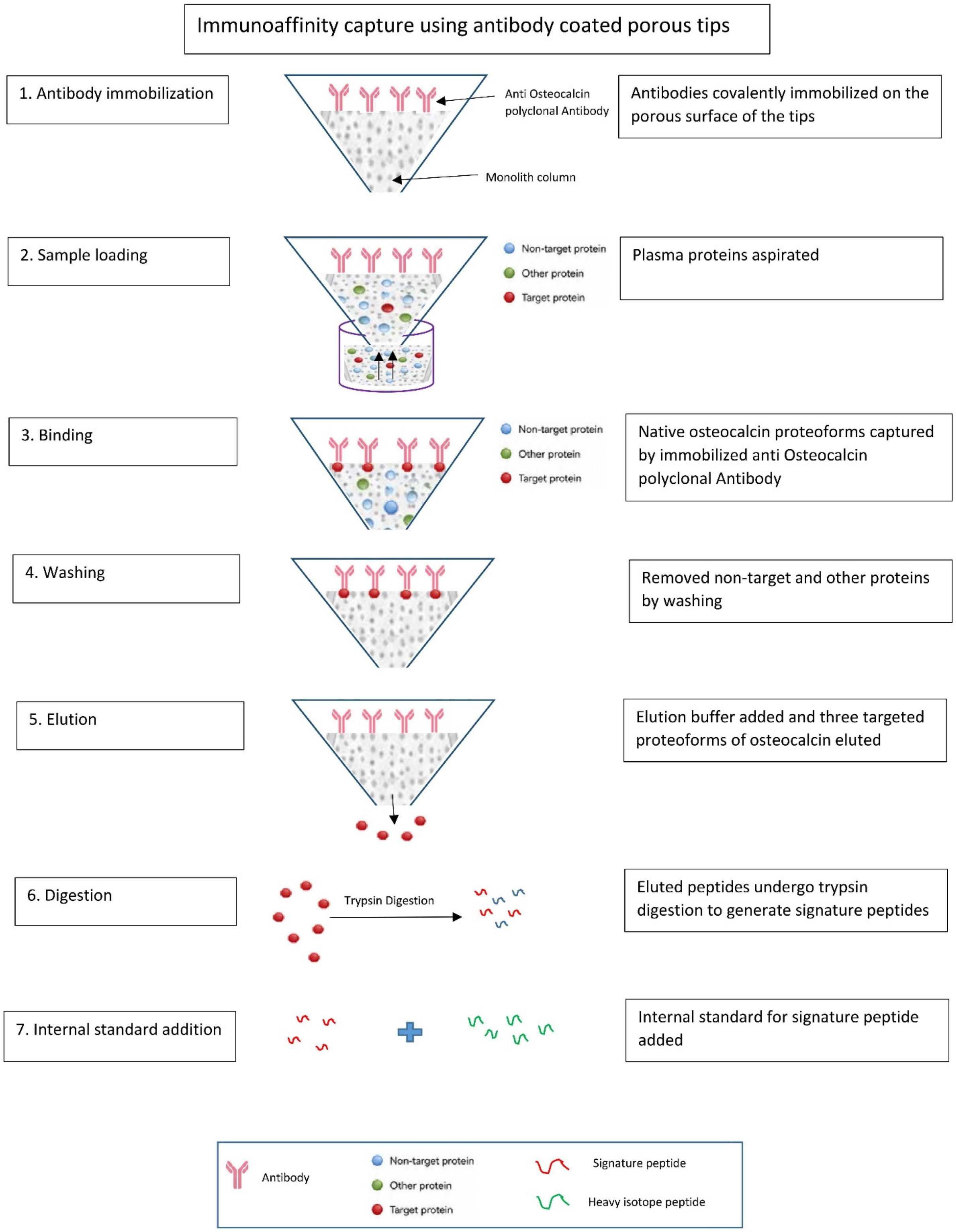

Figure S1. Immunoaffinity capture using antibody coated porous tips
